# Alterations in Gut Microbial Communities Across Anatomical Locations in Inflammatory Bowel Diseases

**DOI:** 10.3389/fnut.2021.615064

**Published:** 2021-02-26

**Authors:** Youlian Zhou, Yan He, Le Liu, Wanyan Zhou, Pu Wang, Han Hu, Yuqiang Nie, Ye Chen

**Affiliations:** ^1^Guangdong Provincial Key Laboratory of Gastroenterology, Department of Gastroenterology, Nanfang Hospital, Southern Medical University, Guangzhou, China; ^2^Department of Gastroenterology, Guangzhou Digestive Disease Center, Guangzhou First People's Hospital, School of Medicine, South China University of Technology, Guangzhou, China; ^3^State Key Laboratory of Organ Failure Research, Division of Laboratory Medicine, Zhujiang Hospital, Southern Medical University, Guangzhou, China; ^4^Shenzhen Xbiome Biotech Co. Ltd., Shenzhen, China

**Keywords:** gut microbiota, Crohn's disease, ulcerative colitis, disease location, *Gardnerella*, *Fusobacterium*

## Abstract

We previously discovered that gut microbiota can serve as universal microbial biomarkers for diagnosis, disease activity assessment, and predicting the response to infliximab treatment for inflammatory bowel diseases (IBD). Much still remains unknown about the relationship between alterations in gut microbiota and IBD affected bowel region, in particular in the case of ulcerative colitis (UC) and colonic Crohn's disease (cCD) without endoscopic and biopsy data. In the current study gut microbiota from a population in China was found to be distinct from that of the Western world [Human Microbiome Project (HMP) data]. Furthermore, both gut microbiota greatly differed from microbiota of other anatomical locations (oral, skin, airway, and vagina), with higher alpha-diversity (Chinese gut > HMP gut > oral microbiome > airway microbiome > skin microbiome > vaginal microbiome), and marked differences in microbiome composition. In patients with IBD in China, UC was characterized by the presence of *Gardnerella*, while cCD was characterized by the presence of *Fusobacterium*. Moreover, gut microbiota, such as *Gardnerella* and *Fusobacterium*, may be potential biomarkers for identifying UC from cCD. Together, this study revealed crucial differences in microbial communities across anatomical locations, and demonstrated that there was an important association between IBD affected bowel region and gut microbiota.

## Introduction

Inflammatory bowel diseases (IBD) involves ulcerative colitis (UC) and Crohn's disease (CD). The chronic course of these diseases can lead to complications, such as narrowing of the gastrointestinal tract, fistulas, abscesses, toxic megacolon, bowel perforation or obstruction, and even carcinogenesis. IBD is common in Europe and North America, but the IBD incidence in China has increased in the past decade (1–3), and it has gradually emerged as the main cause of digestive system diseases and chronic diarrhea. In clinical practice, differential diagnosis of UC and CD is based primarily on clinical manifestation, endoscopy, and histopathological features. However, a definitive diagnosis of UC or CD can be difficult to make, especially in the case of UC and colonic CD (cCD) without endoscopic and biopsy data. Therefore, the identification of biomarkers for distinguishing UC from CD, particularly UC from cCD, is clinically relevant.

Accumulating evidence suggests the gut microbiota has a significant effect on the onset and progression of IBD (4). In patients with IBD, the fecal microbiota diversity is decreased compared to healthy controls (4). Furthermore, major alterations of intestinal microbial diversity in treatment-naive CD are strongly associated with disease status (5–7). Frequent features of patients with IBD, which could distinguish these individuals and healthy subjects, include decreased bacterial diversity, reduced abundance of several communities from the phylum *Firmicutes*, and increased abundance of *Gammaproteobacteria* (8–13). However, most published studies describing intestinal microbiota in IBD are based on western populations, whose genetic history, ethnic background, geographical environment, dietary habits, and lifestyle are distinct from those in the Asia-Pacific region. Published reports on gut microbiota from Chinese patients with IBD in China are limited (14–16), and mainly target to specific bacterial populations. However, some crucial actors in the gut microbiota imbalance of IBD may not yet be known. Our previous study discovered that gut microbiota could serve as universal microbial markers to facilitate diagnosis, activity assessment, and predicting the response to infliximab treatment for IBD (17).

In the current study, the possibility of a universal microbial signature for IBD affected bowel region is explored. In addition, the microbiota of Chinese and Human Microbiome Project (HMP) populations from different anatomical locations are compared based on the sequences they contain.

## Materials and Methods

### Ethical Statement

Ethical approval was obtained from the Ethical Committee of Nanfang Hospital, Southern Medical University (NHMEC2013-081). And all the subjects included in this study were provided with written consent.

### Patients and Samples

Seventy-two patients with CD (39 males, 33 females, mean age 32 years) and 51 patients with UC (28 males, 23 females, average age 42 years old) (Supplementary Table 1) from the Gastroenterology Department in Nanfang Hospital, Southern Medical University in South China were recruited to the study along with 73 healthy volunteers aged 20–55 (gender- and age-matched to the patients) (Supplementary Figure 1). Exclusion criteria included age <18 years, prior IBD treatment, use of probiotics or antibiotics within 1 month, microbial-related chronic diseases such as metabolic diseases (diabetes, hypertension, obesity, metabolic syndrome), cardiovascular diseases, chronic kidney disease, liver diseases, autoimmune diseases, allergic disorders, neuropsychiatric disorders and cancer, and currently pregnant or lactating.

Fecal samples were collected from recruited individuals prior to treatment, who were newly diagnosed and were not received any IBD treatments including anti-inflammatory drugs, immune system suppressors, biologics, antibiotics or surgery, and were processed in <2 h to prevent the exposure of strictly anaerobic bacteria to oxygen. Feces were immediately frozen and kept at −80°C.

### Differential IBD Diagnosis

UC and CD diagnoses adhered to Lennard-Jones criteria (18). Consistent with the Montreal criteria (19), UC was subdivided into extensive UC (pancolitis, E3), left-sided colitis (E2), and ulcerative proctitis (E1) based on disease severity; CD location was classified as ileum CD or colonic CD.

### Extraction of Fecal Genomic DNA

Total DNA from stool samples was extracted with the TIANGEN Stool DNA Kit (TIANGEN Biotech, Beijing) as previously described (20). Concentrations of DNA were determined by Agilent 2100 Bio-analyzer (Calipe Driven, G2939A, Germany), and samples were kept at −20°C prior to PCR analysis.

### PCR Amplification, Illumina Sequencing, and Bioinformatics Analysis

PCR amplification of bacterial 16S rRNA V4 fragments was performed by using barcoded V4-515F 5′-GTGCCAGCMGCCGCGGTAA-3′ and V4-806R 5′-GGACTACHVGGGTWTCTAAT-3′ primers. The PCR amplification conditions were done as previously described (17). A QIAquick Gel Extraction Kit (Qiagen) was used to purify the PCR products. Libraries were sequenced at the Beijing Genomic Institute (Shenzhen, China) by MiSeq (Illumina) with 250-bp paired-end reads according to the manufacturer's instructions.

After removing sequences containing ambiguous bases or mismatches in the primer regions, paired-end sequences were overlapped according to BIPES (21) protocol. A total of 693,788 sequences were acquired from 199 samples, with an average of 2,560 ± 1,249 (SD) sequences per sample. Chimeras were removed with UCHIME using *de novo* mode (parameters: –minchunk20 –xn 7 –noskipgaps 2) (22).

All samples were normalized to 1,005 sequences per sample and uploaded to QIIME (version 1.80) for downstream analyses (23). UCLUST was used to cluster sequences using closed-reference operational taxonomic unit (OTU) picking against the Greengenes database (version gg_13_8) with identity parameter set to 0.97 (24). Phylogenetic diversity (PD) whole-tree values were calculated to evaluate α-diversity, and UniFrac distance was applied to analyze β-diversity. Differentially abundant features were identified using linear discriminant analysis effect size (LEfSe) (25). Random forest (RF) classification models were conducted using the R package ranger. Models were calculated using the area under the curve (AUC) in the receiver operating characteristic (ROC) analysis.

### Human Microbiome Project (HMP) Database Analysis

HMP datasets were downloaded from http://hmpdacc.org/HMQCP/ and the V4 regions from all sequences were selected for comparison. HMP data and IBD data were combined for downstream analyses.

### Quantitative PCR Assays Targeting Bacterial 16S rRNA

The bacteria selected for reverse-transcription-quantitative-PCR (qPCR) assays were *Gardnerella, Fusobacterium nucleatum, Lactobacillus*, and *Bifidobacterium* and the respective qPCR primers are listed in Supplementary Table 2. Each qPCR comprised 10 μL TaKaRa Premix Taq, 2 μL template DNA, 0.4 μL each primer (10 μM), and 7.2 μL double-distilled H_2_O and then following: 95°C for 30 s, then 40 cycles of 95°C for 5 s, 55°C for 30 s, and 72°C for 30 s (Roche LightCycle^@^ 480 II system). DNA copy was expressed by the relative cycle threshold at which DNA for each target relative to the cycle threshold at which “universal bacterial” DNA (16s) using formula 2^−Δ*Ct*^ (relative fold difference compared to the universal 16s) (26).

### Statistical Analysis

Statistical analysis of the diversity indices was conducted using R (version 3.0.2) by Wilcoxon test and FDR (False Discovery Rate) adjusted. qPCR analysis between UC and cCD was performed in GraphPad Prism 6 (San Diego, USA) by *t* test (two-tailed). *P*-values < 0.05 (*p* < 0.05) were considered significant.

## Results

### Microbial Communities Vary Between Populations and Anatomical Locations

**Figure 1:**
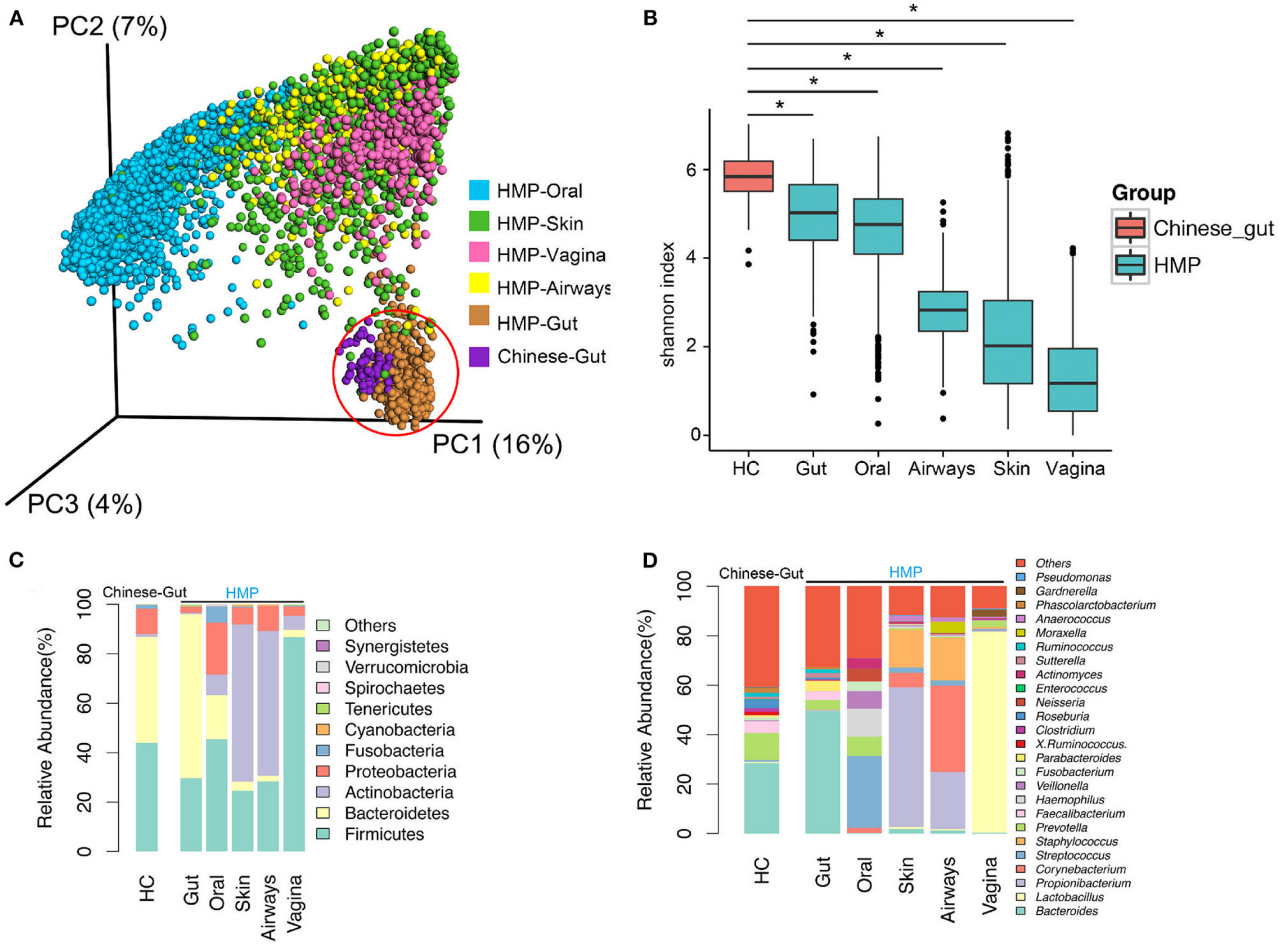
Diversity and abundance of bacteria from the gut and other anatomical locations (oral, skin, airway, and vagina) obtained from healthy subjects in China and Human Microbiome Project (HMP) data. **(A)** Bacterial β-diversity, illustrated by principal coordinate (PC) plots of unweighted UniFrac distance. PC1, PC2, and PC3 captured most of the similarities in microbiota, as shown by percentages. **(B)** Bacterial α-diversity according to the Shannon diversity index. Bacterial diversity differed significantly between a healthy population from China and HMP samples. **(C)** Microbial composition at the phylum level. **(D)** Microbial structure at the genus level. HC, healthy controls. *, *p* < 0.05.

HMP data from 6,885 samples (445 airway, 3,726 oral, 437 gut, 1,713 skin, and 564 vaginal) from healthy American subjects were included in analyses. Principal coordinates analysis (PCoA) revealed that compared to oral, skin, vaginal, and airway microbiota, gut microbiota from the 73 healthy population in China was much more similar to the gut microbiota of the western population (HMP), although the two gut populations were distinct (Figure 1A). Bacterial α-diversity and relative abundance of gut microbiota are shown in Figures 1B–D. Gut bacterial α-diversity was significantly higher in the population of healthy Chinese subjects compared to the western population, and microbiota diversity varied depending on anatomical location (Chinese gut > HMP gut > oral microbiome > airway microbiome > skin microbiome > vaginal microbiome) (*p* < 0.05; Figure 1B). In terms of microbiome composition, *Bacteroidetes* and *Bacteroides* were enriched in gut samples, while there were more *Fusobacteria, Fusobacterium, Streptococcus, Proteobacteria, Prevotella*, and *Veillonella* in oral samples. Both skin and airway samples had increased abundance of *Actinobacteria* and *Staphylococcus*. *Firmicutes*, one of the most important phyla, and *Lactobacillus* were highly enriched in vaginal samples (Figures 1C,D). Compared with western gut samples (HMP), the gut samples from healthy Chinese subjects contained more *Firmicutes* and *Proteobacteria*, but fewer *Bacteroidetes* (Figure 1C). Comparison of bacterial communities at the genus level revealed fewer *Bacteroides* and more *Prevotella* in samples from Chinese subjects than in western samples (Figure 1D).

### Distinctive Bacterial Communities in CD, UC, and Healthy Controls

72 CD, 51 UC, and 73 healthy controls from south China were recruited in the gut microbial analysis. The clinical activity parameters (Supplementary Table 1) including CRP (36.56 ± 42.03 vs. 20.30 ± 41.99 mg/L), ESR (40.03 ± 26.26 vs. 21.65 ± 18.20 mm/h), and fecal calprotectin (2202.99 ± 3652.87 vs. 1332.26 ± 1424.49 mcg/g) were also collected in CD and UC, respectively. The median CDAI score was 258.62 in CD, and the median Mayo score was 8 in UC, which indicated the active diseases in the enrolled IBD patients.

**Figure 2:**
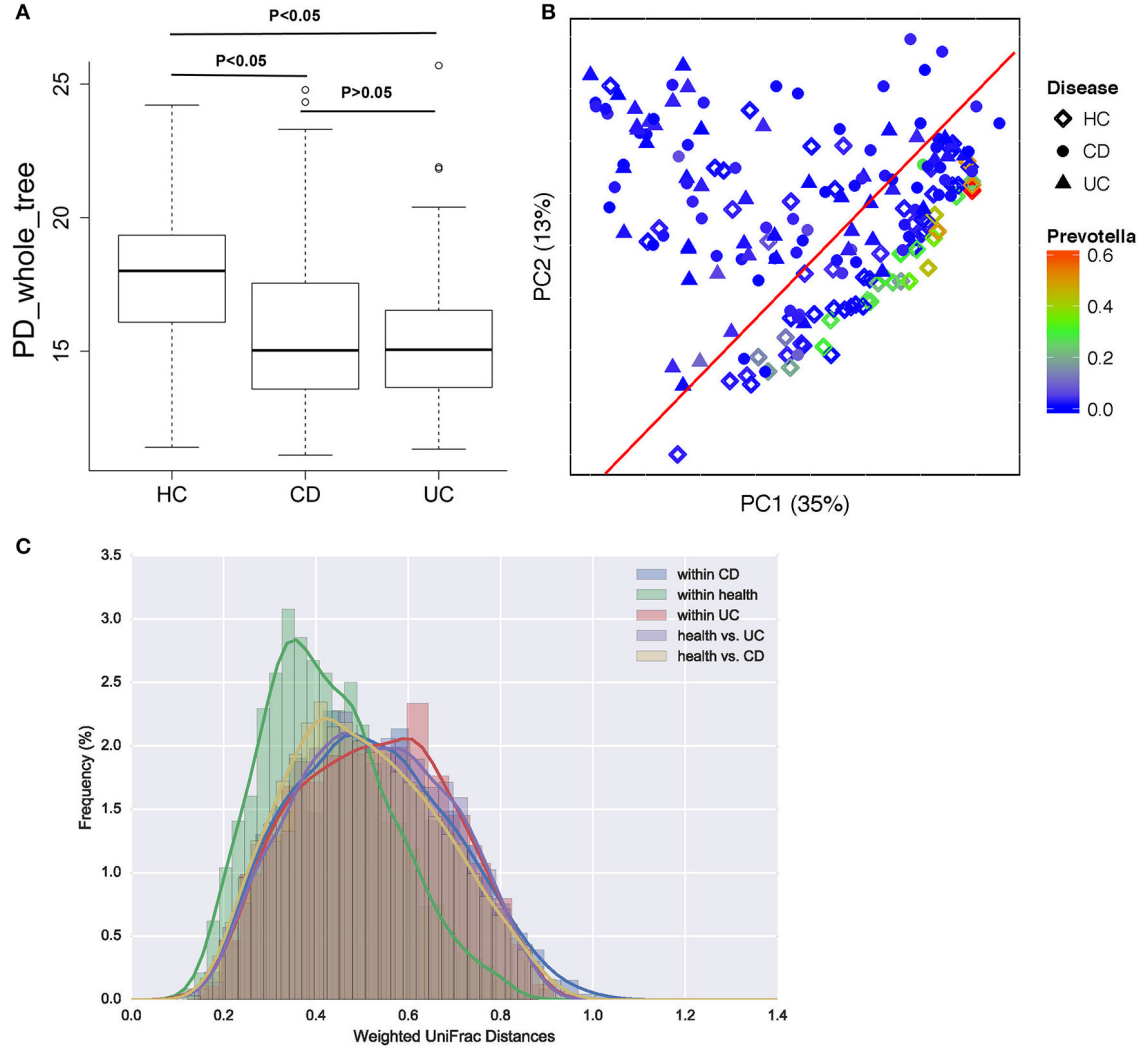
Bacterial diversity in patients with CD or UC and healthy controls (HC). **(A)** Bacterial α-diversity was measured by the PD whole tree value. **(B)** Microbial β-diversity shown by principal coordinate plot of the UniFrac distance across UC, CD and HC, with each sample colored by *Prevotella*, which is more enriched in healthy populations. Each group is defined by a different shape (diamond = HC, circle = CD, triangle = UC). **(C)** Heterogeneity of gut microbiota in weighted UniFrac distance within diseases and health.

A total of 693,788 sequences were acquired from 196 samples, with a mean of 2,560 ± 1,249 sequences per sample. In patients with CD and UC, especially those with UC, α-diversity indices were markedly reduced compared to healthy controls. Microbial diversity was slightly reduced in patients with UC relative to those with CD, but this difference was not significant (Figure 2A). Gut microbiota can be divided into three enterotypes based on three dominant bacteria clusters including *Prevotella* (27), Consequently, bacterial β-diversity was determined using PCoA and the UniFrac distance across samples from patients with IBD and healthy control samples, with each sample colored by *Prevotella*. β-Diversity differed significantly between IBD and healthy controls (ANOSIM test, *p* = 0.001), but no significant difference was observed between samples from patients with CD and those with UC (Figure 2B). The weighted UniFrac distance measured the heterogeneity of gut microbiota within diseased and health (Figure 2C), and indicated that the microbial community varied more in healthy populations than in diseased populations.

**Figure 3:**
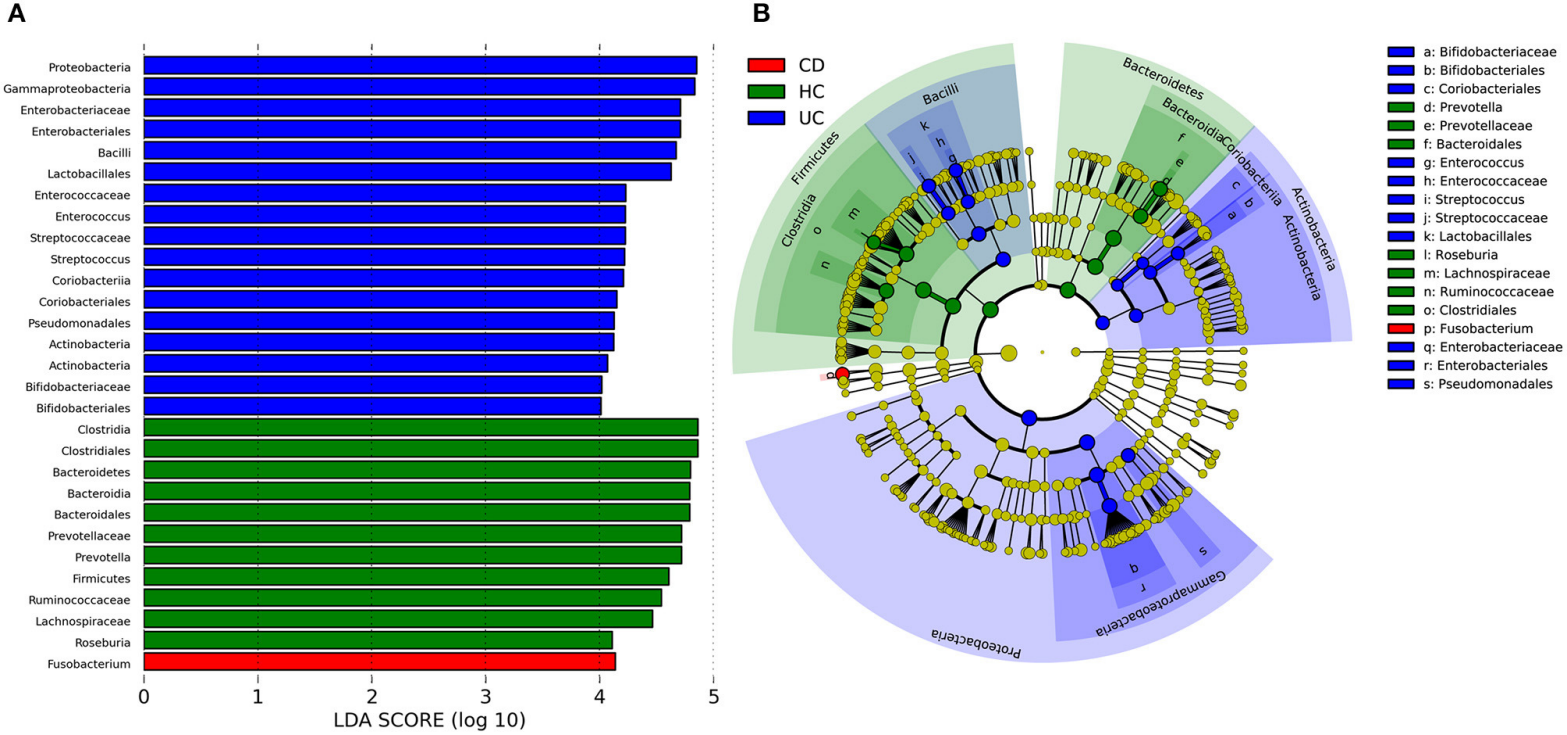
Discriminative taxa determined by LEfSe between healthy controls (HC) and IBD groups. **(A)** Taxa listed according to LDA values, including LDA value of each significantly discriminative taxon. **(B)** Cladogram showed discriminative patterns in taxonomic lineages. LDA cutoff was set to 3.5.

To identify some key differences of intestinal microbial communities between UC and CD, IBD-associated bacterial biomarkers were further explored. UC was characterized by the presence of *Actinobacteria, Proteobacteria, Bacilli, Bifidobacteriaceae, Bifidobacteriales, Coriobacteriales, Enterococcus, Enterococcaceae, Streptococcus, Steptococcaceae, Lactobacillales, Enterobacteriaceae, Enterobacteriales*, and *Pseudomonadales*. CD was characterized by the presence of *Fusobacterium*, while healthy controls (HC) were predominantly enriched in *Prevotella, Prevotellaceae, Bacteroidales, Roseburia, Lachnospiraceae, Ruminococcaceae*, and *Clostridiale*s (Figure 3).

### Identification of Lesion Location in UC and CD by Microflora Biomarker

**Figure 4:**
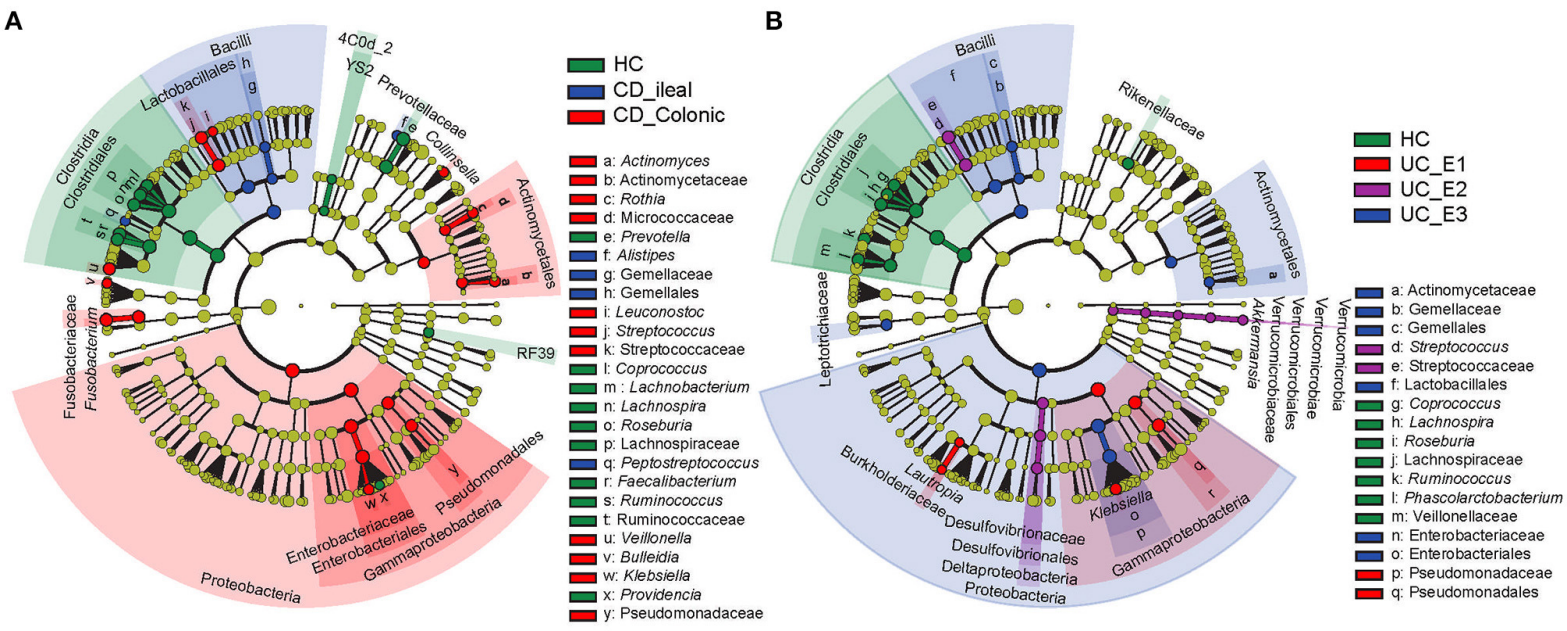
Gut microbial biomarkers for disease location identified using LEfSe. Location classification was determined for CD **(A)** and UC **(B)**. CD was classified as ileal or colonic, and UC was classified as extensive UC (pancolitis, E3), left-sided (distal) UC (E2), or ulcerative proctitis (E1). HC, healthy controls.

Using the Montreal classification (19), CD was subdivided into ileal CD (*N* = 24, 33.33%) and colonic CD (cCD, *N* = 48, 66.67%) based on the extent of the disease. UC was also divided into extensive UC (pancolitis, E3, *N* = 27, 52.94%), left-sided UC (distal UC, E2, *N* = 14, 27.45%), and ulcerative proctitis (E1, *N* = 10, 19.61%) (Supplementary Table 1). LEfSe revealed that more bacterial biomarkers were found for cCD than for ileal CD. *Actinomyces, Actinomycetaceae, Rothia, Micrococcaceae, Leuconostoc, Streptococcus, Streptococcaceae, Veillonella, Bulleidia, Klebsiella*, and *Pseudomonadaceae*, which mainly belong to the phylum *Proteobacteria* and order *Actinomycetales*, were enriched in cCD, while *Alistipes, Gemellaceae, Gemellales*, and *Peptostreptococcu*s were more abundant in ileal CD (Figure 4A). For UC, *Pseudomonadaceae* and *Pseudomonadales* were enriched in E1, while their abundance was much lower in E2 and E3. *Streptococcus and Streptococcaceae* were predominant in E2, and *Actinomycetaceae, Gemellaceae, Gemellales, Enterobacteriaceae*, and *Enterobacteriales* were enriched in E3 (Figure 4B).

### Identification of Key Microbial Phenotypes Responsible for Differentiation Among UC, Colonic CD, and Non-colonic CD

**Figure 5:**
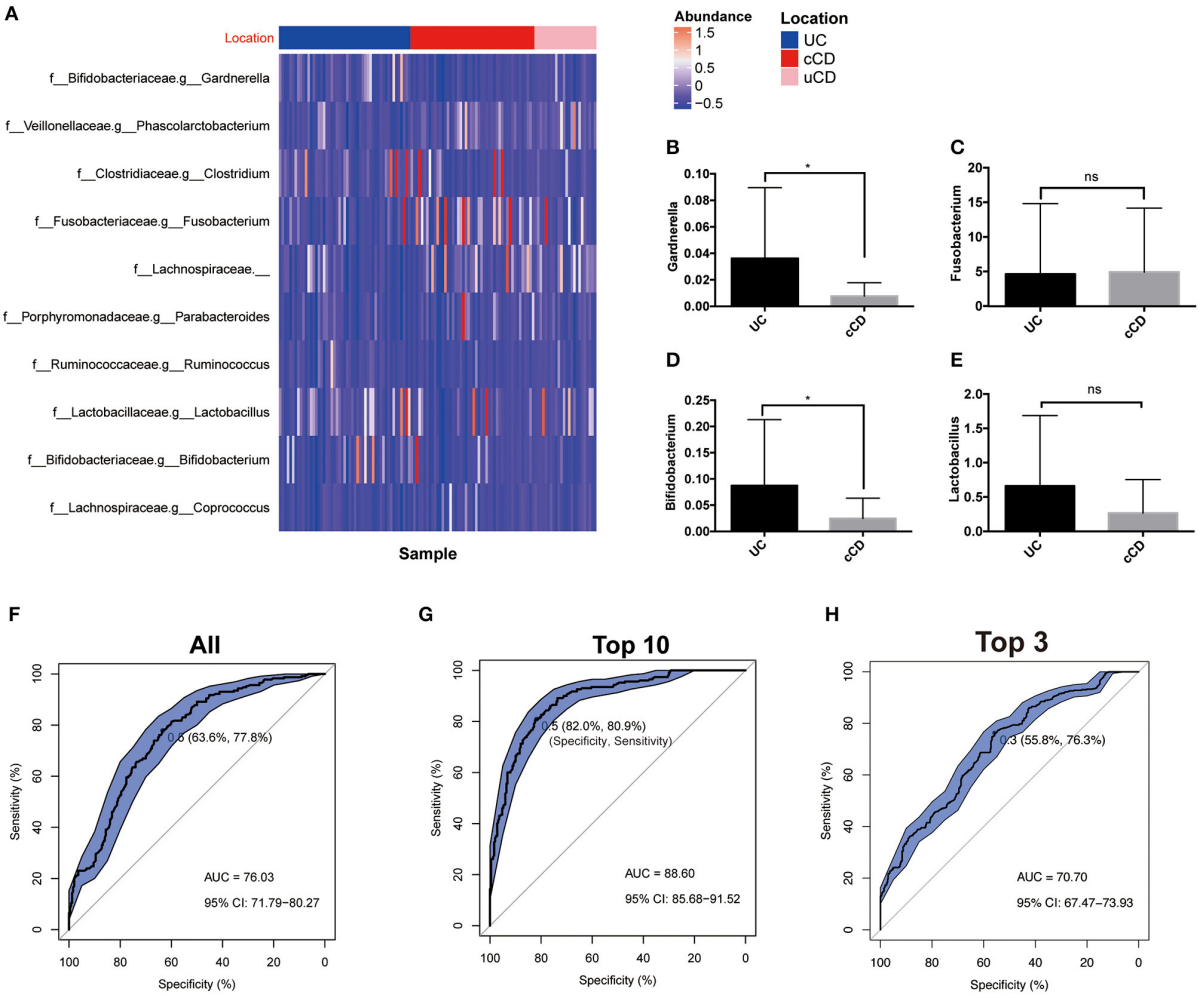
**(A)** Gut microbiome profiles performed well for classifying IBD affected bowel region (UC, *N* = 51; cCD, *N* = 48). **(B–E)** Bacterial quantification targeting *Gardnerella, Fusobacterium nucleatum, Bifidobacterium*, and *Lactobacillus* quantified by qPCR (UC, *N* = 20; cCD, *N* = 21); **(F–H)** Receiver operating characteristic (ROC) plot with area under the curve (AUC) by all taxa in genus level **(F)**, top 10 taxa **(G)** and top 3 including *Gardnerella* and *Fusobacterium* **(H)**. cCD, colonic Crohn's disease; nCD, non-colonic Crohn's disease; UC, ulcerative colitis. *, *p* < 0.05.

In clinical practice, differentiation of UC from CD is consistently difficult, especially without endoscopy and biopsy data. To establish whether gut microbiota can serve as biomarkers for disease phenotype, OTUs were identified and determined to be significantly different in UC, colonic CD (cCD), and non-colonic CD (uCD). *Gardnerella* was significantly more enriched in UC, while *Fusobacterium* was increased in cCD (Figure 5A). To further verify the bacterial sequencing observations, quantification of *Gardnerella, Fusobacterium nucleatum, Lactobacillus*, and *Bifidobacterium* from 20 UC and 21 cCD patients' fecal samples were conducted by qPCR assay. Both *Gardnerella and Bifidobacterium* were also significantly increased in UC compared to cCD, while there was no significant difference in *Fusobacterium nucleatum* between UC and cCD (Figures 5B–E). To further address whether there are some taxa to be used to help predict UC from cCD, we performed a random forest trained prediction model. The use of total gut microbiota at the genus level presented the 76.3% accuracy to distinguish UC from colonic CD (Figure 5F), while the accuracy was as high as 88.6% when 10 taxa including *Fusobacterium, Gardnerella, Odoribacter, Holdemania, Ruminococcus, Sneathia, Paraprevotella, Lactobacillus*, and *Bacteroidales_S24-7* were used in the prediction model (Figure 5G). However, the AUC was only 70.7% when using top 3 taxa containing *Gardnerella* and *Fusobacterium* (Figure 5H). These findings indicate that gut microbiota, such as *Gardnerella* and *Fusobacterium*, may be potential biomarkers for identifying disease location in patients with IBD.

## Discussion

Microbiota composition is known to depend on geographic origin (28), dietary habits (29, 30), obesity (31), ethnic background and genetic characteristics (32), and/or other lifestyle factors. However, there is limited information about differences in microbiota composition among human populations outside of Europe and the US. In this study, population-based variation was revealed to be an important factor in microbiota diversity and composition across anatomical locations (gut, airway, oral, skin, and vagina) in healthy populations, as demonstrated by differences between populations from China and the HMP. To our knowledge, this study is the first to identify distinctive gut bacterial communities in healthy populations in China and the Western world.

IBD, including UC and CD, are chronic inflammatory gastrointestinal diseases. Distinctive features of CD are penetrating inflammation, which may involve any part of the digestive tract, while UC usually involves superficial inflammation of the rectum extending to the adjacent mucosa. When IBD is determined, it can be difficult to distinguish CD from UC due to overlap in histologic and endoscopic features of the two conditions. Moreover, in clinical practice, the location of UC is graded according to invasive colonoscopy, and CD is graded according to gastroscopy, capsule endoscopy, and even colonoscopy. The current study aimed to identify non-invasive biomarkers for distinguishing UC from CD, especially UC from colonic CD (cCD).

Increasing attention has been paid to the gut microbiome in the pathogenesis and management of IBD (13, 33–36). For example, adherent-invasive *Escherichia coli* (*E. coli*) occurred more frequently in patients with CD than in non-IBD controls (37, 38). Martinez-Medina et al. (39, 40) reported a lower prevalence of adherent-invasive *E. coli* in patients with colonic-CD (25% of colonic samples and 50% of ileal samples) than in patients with ileal-CD (58.3% of colonic samples and 66.7% of ileal samples). Differences in the genetic predisposition and immunoreactivity of gut microbiota based on lesion location have also been reported (41), indicating that the gut microbiome pattern may present as variability in disease phenotype. Previous researches did not determine the location of CD or distinguish between UC and cCD. It is important that differences in intestinal microbiota are identified in relation to disease phenotype as they may provide information about potential biomarkers for conditions such as IBD.

Consistent with previous studies on colonic mucosa-associated bacterial microbiota (42), diversity in the study cohort with IBD was reduced compared to controls, with a greater reduction in diversity observed in patients with UC (not statistically significant compared to CD). Detailed compositional changes in the gut microflora of patients with IBD were investigated at different bacterial taxonomic levels. At the genus level, *Fusobacterium* was more abundant in patients with CD by 16S sequencing, while there was no marked difference in Fusobacterium by qPCR quantification. As it is difficult to address microbiota in the species level especially in the strain level by bioinformatic analysis after 16S sequencing. Furthermore, *Fusobacterium* in the genus level was divided into many different species and strains including *Fusobacterium nucleatum*. So it is not surprise in the difference between *Fusobacterium* tested by 16S sequencing and *Fusobacterium nucleatum* verified by qPCR. However, it is worth noting that *Fusobacterium nucleatum* was reported to contribute to colorectal carcinoma by promoting a beneficial microenvironment for carcinoma progression in UC (43) and has also been suggested as a biomarker for IBD (44). The presence of *Fusobacterium* has been associated with long-term complications in patients with CD, such as colorectal cancer and fistula. Bacterial species from the genera *Prevotella, Roseburia*, and *Clostridiales* (typically indigenous) were significantly lower in patients with IBD, while *Enterococcus* and *Streptococcus* (typically pathogens or opportunistic pathogens) were markedly higher. *Gardnerella* is a genus of Gram-variable, facultatively anaerobic bacteria, of which *Gardnerella vaginalis* (*G. vaginalis*) is the only species. *G. vaginalis* is considered to be main cause of bacterial vaginosis and is present in a dispersed form or as a biofilm. Schilling et al. (45) discovered that the positive rate of *G. vaginalis* biofilm were increased in patients with UC and CD compared with healthy individuals. Moreover, a link was also found between steroid-refractory or -dependent disorder and *G. vaginalis* biofilms. In the current study, *Gardnerella* was significantly more enriched in fecal samples from patients with UC. These microbiota composition data indicate that *Gardnerella* and *Fusobacterium* may be potential biomarkers for identifying CD and UC.

There are several limitations to the current study. First, despite promising correlations between microbial alterations and disease phenotypes, a causative role of the variation in microbiota has not been determined, and our understanding of the dynamic role of gut microbiota in the IBD affected bowel region remains incomplete. In addition, research on new-onset IBD suggests that the decreased microbial diversity noted in adults may not be found in pediatric or elderly patients. Despite these shortcomings, the present study provides a comparison of bacterial communities between healthy populations in China and the Western world (HMP data), and also the microbial dysbiosis in patients with IBD in China. Moreover, the study aimed to identify microbial biomarkers of lesion location in patients with CD and UC, especially the key phenotypes responsible for differentiating UC, colonic CD (cCD), and non-colonic CD, which would further define clinical guidelines for IBD treatment. The study conceptually demonstrates the potential to use the gut microbiome to aid in UC and CD diagnosis and is therefore of significant clinical value in the management of IBD. Future studies are necessary to characterize the functions of gut flora in IBD and attempt to manipulate the commensal microflora in patients with IBD.

## Data Availability Statement

The data presented in the study are deposited in the ENA repository under accession number PRJEB22028.

## Ethics Statement

The studies involving human participants were reviewed and approved by the Ethical Committee of Nanfang Hospital, Southern Medical University (NHMEC2013-081). The patients/participants provided their written informed consent to participate in this study.

## Author Contributions

YZ: design of the study, recruitment of patients, statistical analysis and interpretation of the data, and drafting of the article. YH: bioinformatics analysis and interpretation of the data. LL: interpretation of the data and revision of the article. WZ and PW: recruitment of patients and statistical analysis. HH: assisting part of bioinformatics analysis. YN: interpretation of the data and revision of the article. YC: concept and design of the study, interpretation of the data, and revision of the article. All authors contributed to the article and approved the submitted version.

## Conflict of Interest

The authors declare that the research was conducted in the absence of any commercial or financial relationships that could be construed as a potential conflict of interest.

## Supplementary Material

Supplementary Figure 1 Study design of this study.

Supplementary Table 1 Baseline clinical characteristics of the subjects.

Supplementary Table 2 Primers used for RT-PCR in this study.
