# Supplementary figures and images for: Alterations in Gut Microbial Communities Across Anatomical Locations in Inflammatory Bowel Diseases
Source: Front Nutr. 2021 Feb 26;8:615064. doi: 10.3389/fnut.2021.615064 (PMC7952524; doi:10.3389/fnut.2021.615064)

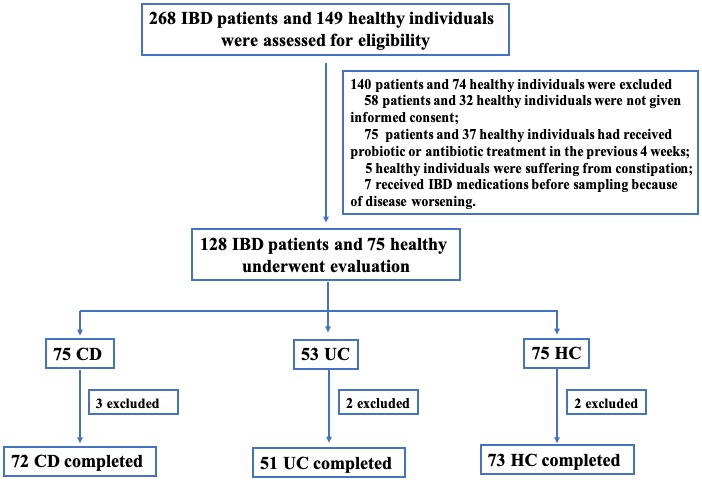

Supplement: Supplementary Figure 1 — Study design of this study. [file Image_1.JPEG]
