# Supplementary material for: Alterations in Gut Microbial Communities Across Anatomical Locations in Inflammatory Bowel Diseases
Source: Front Nutr. 2021 Feb 26;8:615064. doi: 10.3389/fnut.2021.615064 (PMC7952524; doi:10.3389/fnut.2021.615064)
Supplement: Supplementary Table 1 — Baseline clinical characteristics of the subjects. [file Table_1.docx]

**Table 1** Baseline clinical characteristics of the subjects.

|  | | **HC (n=73)** | | **CD (n=72)** | | **UC (n=51)** |
| --- | --- | --- | --- | --- | --- | --- |
| Sex (female/male) | 30/43 | | 33/39 | | 23/28 | |
| Age (years; mean±SD) | 30.07±6.36 | | 31.81±12.668 | | 41.75±14.362 | |
| CDAI score (median) | NA | | 258.62 | | NA | |
| Mayo score (median) | NA | | NA | | 8.00 | |
| CRP (mg/L; mean±SD) | NA | | 36.56±42.03 | | 20.30±41.99 | |
| ESR (mm/h; mean±SD) | NA | | 40.03±26.26 | | 21.65±18.20 | |
| Fecal calprotectin (mcg/g; mean±SD) | NA | | 2202.99±3652.87 | | 1332.26±1424.49 | |
| Extent of disease [n (%)] |  | |  | |  | |
| UC Proctitis (E1) | NA | | NA | | 10 (19.61%) | |
| Left-sided (E2) | NA | | NA | | 14 (27.45%) | |
| Pancolitis (E3) | NA | | NA | | 27 (52.94%) | |
| Colonic CD | NA | | 48 (66.67%) | | NA | |
| Non-colonic/ ileal CD | NA | | 24 (33.33%) | | NA | |

HC, healthy controls; CD, Crohn’s disease; UC, ulcerative colitis; SD, standard deviation; NA, not available/applicable; CDAI, Crohn’s Disease Activity Index; CRP, C--reactive protein； ESR, erythrocyte sedimentation rate.
