# Supplementary material for: Alterations in Gut Microbial Communities Across Anatomical Locations in Inflammatory Bowel Diseases
Source: Front Nutr. 2021 Feb 26;8:615064. doi: 10.3389/fnut.2021.615064 (PMC7952524; doi:10.3389/fnut.2021.615064)
Supplement: Supplementary Table 2 — Primers used for RT-PCR in this study. [file Table_2.DOCX]

Table S2: Primers used for RT-PCR in this study.

| Target bacteria | Primer and Sequence (5’to 3‘) |
| --- | --- |
| *Gardnerella* | F: AAGCGCAGAAGGACGAAAAG |
|  | R: TGCTACACGATCCACACCTAAC |
| *Fusobacterium nucleatum* | F: TGGTCAGGATGAGAAATC |
|  | R: ATCGCATGGATACTAACA |
| *Bifidobacterium* | F: TCTCGCTACTCATGTCTG |
|  | R: CACTGGTCTAGTGGTTCC |
| *Lactobacillius* | F: AGCAGTAGGGAATCTTCCA |
|  | R: CACCGCTACACATGGAG |
| Universal bacterial 16S | F: GGGAATATTGCACAATGG |
|  | R: TGAGCAAAGGTATTAACTTTAC |
